# Epidural analgesia and cesarean delivery in multiple sclerosis post-partum relapses: the Italian cohort study

**DOI:** 10.1186/1471-2377-12-165

**Published:** 2012-12-31

**Authors:** Luisa Pastò, Emilio Portaccio, Angelo Ghezzi, Bahia Hakiki, Marta Giannini, Lorenzo Razzolini, Elisa Piscolla, Laura De Giglio, Carlo Pozzilli, Damiano Paolicelli, Maria Trojano, Maria Giovanna Marrosu, Francesco Patti, Loredana La Mantia, Gian Luigi Mancardi, Claudio Solaro, Rocco Totaro, Maria Rosaria Tola, Valeria Di Tommaso, Alessandra Lugaresi, Lucia Moiola, Vittorio Martinelli, Giancarlo Comi, Maria Pia Amato

**Affiliations:** 1 Department of Neurology, University of Florence, Viale Morgagni 85, 50134, Florence, Italy; 2 Multiple Sclerosis Center, S Antonio Abate Hospital, Gallarate, Italy; 3 Multiple Sclerosis Center, S Andrea Hospital, La Sapienza University, Rome, Italy; 4 Department of Neurology, University of Bari, Bari, Italy; 5 Multiple Sclerosis Center, Department of Neurology, University of Cagliari, Cagliari, Italy; 6 Multiple Sclerosis Center, University of Catania, Catania, Italy; 7 MS Center, C Besta National Neurological Institute, Milan, Italy; 8 Department of Neurology, University of Genova, Genova, Italy; 9 Department of Neurology, ASL3 Genovese, Genova, Italy; 10 Department of Neurosciences, University of L’Aquila, L’Aquila, Italy; 11 Department of Neuroscience, University of Ferrara, Ferrara, Italy; 12 Department of Neurosciences and Imaging, University G. d'Annunzio Chieti, Centro Sclerosi Multipla, Chieti, Italy; 13 Scientific Institute University Vita-Salute San Raffaele, Milan, Italy; 14 Don Carlo Gnocchi Foundation, ONLUS, Florence, Italy

**Keywords:** Epidural analgesia, Caesarean delivery, Multiple sclerosis, Pregnancy

## Abstract

**Background:**

Few studies have systematically addressed the role of epidural analgesia and caesarean delivery in predicting the post-partum disease activity in women with Multiple Sclerosis (MS).

The objective of this study was to assess the impact of epidural analgesia (EA) and caesarean delivery (CD) on the risk of post-partum relapses and disability in women with MS.

**Methods:**

In the context of an Italian prospective study on the safety of immunomodulators in pregnancy, we included pregnancies occurred between 2002 and 2008 in women with MS regularly followed-up in 21 Italian MS centers. Data were gathered through a standardized, semi-structured interview, dealing with pregnancy outcomes, breastfeeding, type of delivery (vaginal or caesarean) and EA. The risk of post-partum relapses and disability progression (1 point on the Expanded Disability Status Sclae, EDSS, point, confirmed after six months) was assessed through a logistic multivariate regression analysis.

**Results:**

We collected data on 423 pregnancies in 415 women. Among these, 349 pregnancies resulted in full term deliveries, with a post-partum follow-up of at least one year (mean follow-up period 5.5±3.1 years). One hundred and fifty-five patients (44.4%) underwent CD and 65 (18.5%) EA. In the multivariate analysis neither CD, nor EA were associated with a higher risk of post-partum relapses. Post-partum relapses were related to a higher EDSS score at conception (OR=1.42; 95% CI 1.11-1.82; p=0.005), a higher number of relapses in the year before pregnancy (OR=1.62; 95% CI 1.15-2.29; p=0.006) and during pregnancy (OR=3.07; 95% CI 1.40-6.72; p=0.005). Likewise, CD and EA were not associated with disability progression on the EDSS after delivery. The only significant predictor of disability progression was the occurrence of relapses in the year after delivery (disability progression in the year after delivery: OR= 4.00; 95% CI 2.0-8.2; p<0.001; disability progression over the whole follow-up period: OR= 2.0; 95% CI 1.2-3.3; p=0.005).

**Conclusions:**

Our findings, show no correlation between EA, CD and postpartum relapses and disability. Therefore these procedures can safely be applied in MS patients. On the other hand, post-partum relapses are significantly associated with increased disability, which calls for the need of preventive therapies after delivery.

## Background

The influence of pregnancy in multiple sclerosis (MS) has been extensively assessed. The large Pregnancy In Multiple Sclerosis (PRIMS) study reported that the rate of relapses decreases during the pregnancy, increases during the first trimester of postpartum, then returning to the prepregnancy rate after delivery [1]. A pathophysiological hypothesis for explaining the spontaneous remission of MS during pregnancy is that pregnancy is associated with a decrease in cellular immunity and an increase in humoral immunity, and a shift away from Th1 to Th2 responses; on the contrary, delivery is associated with an inversion of this balance and a shift from Th2 a Th1 responses [1].

Only three factors seem independently predictive of an increase of relapses in the three-month post-partum period: the number of relapses in the year before pregnancy, the number of relapses during pregnancy, and the duration of MS [2]. The possible role of breastfeeding is still under discussion. A recent study suggested a protective role of exclusive breastfeeding on the risk of post-partum relapses [3,4]. However, findings from larger studies PRIMS [1] and other more recent papers [5-7] indicated that the reported association between breastfeeding and a lower risk of relapses may simply reflect a different patient behavior, since patients with a less active disease are more likely to breastfeed.

Few studies have addressed the role of epidural analgesia (EA) and caesareum delivery (CD) in predicting the post-partum disease activity [1]. The PRIMS reported no correlation between postpartum relapses and epidural analgesia [2,8] although in that study this represented a secondary outcome and no detailed analysis is provided.

In a previous multicentric, prospective study on a cohort of pregnant women followed-up in the main Italian MS centers, we assessed the issues of disease-modifying drugs (DMDs) safety [5] during pregnancy and the role of breastfeeding [7]. The study also gathered detailed information on the use of epidural analgesia and type of delivery as well as follow-up data on disease course after delivery. In this further analysis of the same cohort, we aimed at assessing the impact of EA and CD on the post-partum relapse rate and disability, taking into account possible confounders.

## Methods

Between 2002 and 2008, all pregnancies occurring in MS patients diagnosed according to McDonald’s criteria [9] and referred to the participating centers were identified and tracked over the whole gestational period. The 21 participating sites represented the main Italian MS Centers located throughout the entire country. In the present study, we included all the pregnancies resulting in full term deliveries and having a post-partum follow-up duration of at least one year. All the patients were regularly followed-up after the delivery every 6 months and in the case of relapse. Clinical and therapeutic data were gathered by the neurologist using a standardized information form. After the delivery, the neurologist administered a semi-structured interview to each patient dealing with in utero exposures, pregnancy outcomes and breastfeeding (see Additional file 1). Moreover, data on type of delivery were categorized into vaginal, with assistance (use of forceps or ventose) or without assistance, or caesarean; information on epidural analgesia included the reason (analgesia or anesthesia for caesarean delivery). As for disease activity, date of onset and number of relapses in the year prior to conception, during pregnancy and in the year after delivery were recorded. A relapse was defined as the appearance or reappearance of one or more symptoms attributable to MS, accompanied by objective deterioration on neurological examination lasting at least 24 hours, in the absence of fever and preceded by neurological stability for at least 30 days [9]. Disability was also recorded on the Functional Systems and Expanded Disability Status Scale (EDSS) [10] in case of relapses and over the follow-up period. Disability progression was defined as a 1.5 point EDSS increase for baseline score= 0; 1point EDSS increase for baseline scores 1–5.5 and 0.5 point EDSS increase for baseline scores >5.5. The EDSS increase had to be confirmed at 6 and 12 months.

The study was approved by the ethic committee of the University of Florence and a written consent was obtained from all patients.

### Statistical analysis

Group comparison was assessed with Pearson’s |2, Student t and Mann–Whitney U tests, when appropriate.

An annualized relapse-rate was calculated for each trimester in the year before, during and after pregnancy. The relapse –rate in patients who underwent CD and patients who did not, and patients who underwent EA and patients who did not was compared using a 2 (Groups) × 11 (Time – 4 trimesters before conception, 3 trimesters during pregnancy, 4 trimesters after delivery) mixed factorial design, with repeated measures on the second factor. This allows to evaluate differences between the two groups (effect for group), within each group over time (effect for time) and the interaction between group and time (effect for group x time).

Moreover, the patients were grouped in patients with at least one relapse and in patients without relapses in the year after delivery. The impact of CD and EA and other possible predictors of post-partum relapses was assessed through a logistic regression model. The following covariates were entered in the model as possible confounders: age at MS onset, age, disease duration and EDSS at conception (baseline), DMDs before pregnancy, number of relapses in the year before pregnancy and during pregnancy, exclusive breastfeeding. The sample size was not estimated a-priori. However, the sample included in our study would be able to detect a difference of 0.15 between two proportions with a significance level of 0.05 and a power of approximately 0.80. All analyses were performed using the SPSS 18.0 running on Windows (SPSS, Chicago, IL, USA).

## Results

**Figure 1:**
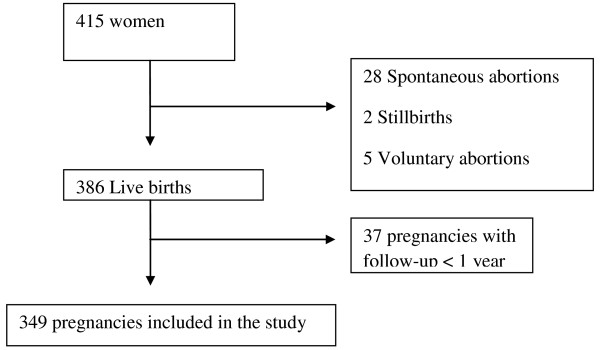
**Flow**-**chart of the pregnancies enrolled in the study.**

During the study period, a total of 423 pregnancies were tracked in 415 women. The last pregnancy included took place on January 2008. Among these, 349 pregnancies resulted in full term deliveries, with a post-partum follow-up of at least one year (Figure 1). Table 1 shows the main demographic and clinical characteristics of the study cohort. No woman was lost to the follow-up. On the whole, CD was applied in 155 patients (44.4%). Sixty-five patients (18.5%) underwent EA (for analgesia in 19, for CD in 46 subjects). There were no differences between either patients with CD or patients with EA in terms of the main demographic and clinical characteristics. A trend was observed towards a higher proportion of treatment with disease modifying drugs before pregnancy in patients with CD (Table 1).

**Table 1:** Characteristics of the study sample

	**Total sample (#349)**	**EA (#65)**	**NEA (#284)**	**p**	**CD(#155)**	**NCD (#194)**	**p**
Age at conception (mean, SD, years)	31.8 (4.7)	32.5 (4.7)	31.7 (4.7)	0.232	31.7 (4.7)	31.8 (4.7)	0.907
Age at onset (mean, SD, years)	24.7 (5.8)	24.6 (6.4)	24.8 (5.7)	0.860	24.5 (6.0)	24.8 (5.7)	0.550
Disease duration at conception (mean, SD, years)	7.1 (4.8)	7.8 (5.9)	6.97 (4.5)	0.279	7.27 (4.8)	7.0 (4.8)	0.689
EDSS at conception (mean, SD)	1.5 (1.0)	1.5 (0.97)	1.5 (0.98)	0.868	1.6 (1.0)	1.4 (0.92)	0.107
Treated with DMDs before pregnancy (#, %)	176 (50.4)	28 (43.1%)	147 (51.8)	0.226	87 (56.1)	89 (45.9)	0.057
Relapses in the year prior to pregnancy (mean, SD)	0.4 (0.7)	0.37 (0.6)	0.39 (0.7)	0.800	0.38 (0.73)	0.39 (0.7)	0.937
Relapses during pregnancy (mean, SD)	0.12 (0.4)	0.1 (0.3)	0.11 (0.3)	0.950	0.15 (0.42)	0.09 (0.29)	0.157
Relapses in the year after the delivery (mean, SD)	0.45 (0.7)	0.35 (0.57)	0.48 (0.76)	0.130	0.47 (0.8)	0.44 (0.67)	0.677
Exclusive breastfeeding (#,%)	162 (46.4)	19 (29.2)	99 (34.9)	0.370	47 (30.3)	71 (36.6)	0.316

*EA*: epidural analgesia.

*NEA*: Not epidural analgesia.

*CD*: caesarean delivery.

*NCD*: not caesarean delivery.

*SD*: Standard Deviation.

*EDSS*: Expanded Disability Status Scale.

*DMDs*: Disease Modifying Drugs.

**Figure 2:**
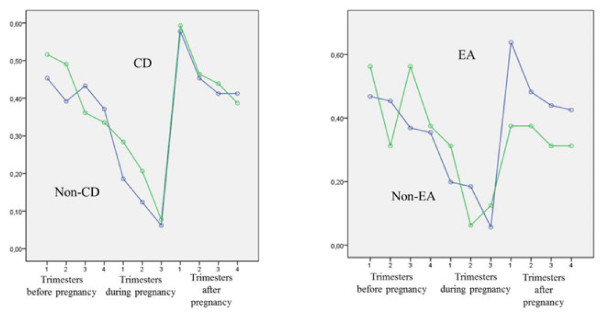
**Annualized relapse**-**rate before**, **during and after pregnancy in patients who underwent CD(left)and patients who underwent EA(right).** CD: Caesarean Delivery, EA: Epidural analgesia.

The annualized relapse-rate in the year before conception, during pregnancy and after delivery is illustrated in Figure 2. On the whole, relapse-rate significantly decreased during pregnancy, particularly in the third trimester, and increased in the post-partum, particularly in the first trimester (effect for time F=6.07-3.25, p<0.001). There were no differences in the mean relapse-rate and in the time-dependent profile of the relapse-rate comparing patients with and without caesarean delivery (effect for group F=0.26, p=0.613; effect for group x time F=0.21, p=0.996), as well as comparing patients with and without epidural analgesia (effect for group F=0.29, p=0.589; effect for group x time F=0.79, p=0.638).

In the year after the delivery, 148 patients (42.4%) experienced at least one relapse (Table 2). Patients with relapses in the post-partum period had a higher EDSS score at conception (1.72 ± 1.11 versus 1.37 ± 0.84: p=0.001), experienced a higher number of relapses in the year prior to pregnancy (0.56 ± 0.84 versus 0.25 ± 0.58: p<0.001) and during pregnancy (0.19 ± 0.46 versus 0.06 ± 0.23; p<0.001). Moreover, the proportion of breastfeeding tended to be lower in patients with relapse (29.1% versus 38.8%: p=0.066).

**Table 2:** **Characteristics of patients with and without post**-**partum relapses**

	**Relapsing (#148)**	**Non relapsing (#201)**	**p**
Age at conception (mean, SD, years)	31.5 (5.0)	32.1 (4.5)	0.241
Age at onset (mean, SD, years)	24.8 (6.1)	24.7 (5.7)	0.930
Disease duration at conception (mean, SD, years)	6.9 (5.0)	7.4 (4.8)	0.355
EDSS at conception (mean, SD)	1.7 (1.1)	1.4 (0.8)	0.001
Treated with DMDs prior to pregnancy (#, %)	70 (47.3)	106 (52.7)	0.315
Relapses in the year prior to pregnancy (mean, SD)	0.56 (0.84)	0.25 (0.59)	<0.001
Relapses during pregnancy (mean, SD)	0.19 (0.46)	0.06 (0.23)	<0.001
EA (#, %)	24 (16.2)	40 (19.9)	0.371
CD (#, %)	62 (41.9)	93 (46.3)	0.416
Breastfeeding (#, %)	43 (29.1%)	78 (38.8%)	0.066

*SD*: Standard Deviation.

*EDSS*: Expanded Disability Status Scale.

*DMDs*: Disease Modifying Drugs.

EA: Epidural Analgesia.

CD: Caesarean Delivery.

In the multivariate analysis, neither CD, nor EA were associated with a higher risk of post-partum relapses. Post-partum relapses were related to a higher EDSS at conception (OR= 1.42; 95% CI 1.11-1.82; p=0.005), a higher number of relapses in the year before pregnancy (OR= 1.62; 95% CI 1.15-2.29; p=0.006) and during pregnancy (OR= 3.07; 95% CI 1.40-6.72; p=0.005). The predictive role of a higher number of relapses in the year before pregnancy and during pregnancy was also confirmed focusing the analysis on the relapse rate in the first trimester post-partum (p< 0.035).

Disability progressed in 42 (12.0%) of patients in the year after delivery and in 96 (27.5%) over the whole follow-up period (mean follow-up period 5.5 ± 3.1 years). In both cases there was no association with CD and EA. The only significant predictor of disability progression was the occurrence of relapses in the year after delivery (disability progression in the year after delivery: OR= 4.00; 95% CI 2.0-8.2; p<0.001; disability progression over the whole follow-up period: OR= 2.0; 95% CI 1.2-3.3; p=0.005).

## Discussion

Approximately two third of patients with MS are women in their childbearing-age and issues related to pregnancy are of crucial importance in the decision making process and counseling. In everyday clinical practice, neurologists often are inquired by the patients on the impact of the pregnancy on the disease course, the safety of disease modifying therapies for both the mother and the child, and the safety and feasibility of perinatal procedures, such as EA, CD and breastfeeding. While the influence of pregnancy on MS course is largely known [1], to date, there is accumulating evidence on the safety of interferon and glatiramer acetate exposure in terms of both maternal and fetal outcomes. Conversely, relatively little is known about the role of EA and CD as predictors of post-partum disease activity, and available information comes mainly from old studies on small samples [8]. An old study pointed to an increased risk of relapses after the administration of bupivacaine greater than 2,5 mg/ml [11]. However, this finding was not confirmed in more recent investigations [12]. The results from our large cohort show that EA, performed in 18.5% of our patients, did not significantly affect post partum relapses or EDSS. Similarly, CD, performed in 44.4% was not associated to a worsening of the MS after the delivery. As observed in the PRIMS study, the risk of post-partum relapses and disability progression was predicted by higher pre-pregnancy and gestational disease activity.

As for CD, it has to be noted that in our sample, the proportion of caesarean deliveries was in the upper limit of the estimates comparing with the general Italian population [5].

In the interpretation of the study results, some possible limitations should be taken into account. Although the 21 participating sites represented the largest Italian MS centers and covered the whole territory, the study population may not be entirely representative of the general population of MS patients and study findings may be affected to some extent by local differences in overall use of EA and CD across the country. Moreover, there is no information on the type and dosages of anesthetic used for EA.

## Conclusions

On the whole, our study provides data useful for patient counseling, showing that EA and CD can be considered safe in female MS patients and adopted when appropriate, without any risk of increased disease activity.

## Abbreviations

MS: Multiple sclerosis; EA: Epidural analgesia; CD: Caesarean delivery; DMDs: Disease-modifying drugs; RR: Relapsing-remitting; ORs: Odds ratios; CIs: Confidence intervals; SD: Standard Deviations; EDSS: Expanded Disability Status Scale.

## Competing interests

**Dr**. **Giannini** has received compensation from Biogen Idec. **Dr**. **Portaccio** serves on a scientific advisory board for Biogen Idec and receives research support from Merck Serono, Biogen Idec, Bayer Schering Pharma, and sanofi-aventis. **Dr**. **Ghezzi** serves on scientific advisory boards for Merck Serono and Teva Pharmaceutical Industries Ltd.; has received speaker honoraria from Merck Serono, Biogen Idec, Bayer Schering Pharma, and Novartis; serves as a consultant for Novartis; and receives research support from Sanofi-aventis, Biogen Idec, and Merck Serono. **Dr**. **Hakiki** receives research support from Novartis and Merck Serono; received funding for travel from Biogen, Sanofi, Novartis, Bayer, Merck Serono. **Dr**. **Pastò** has received compensation from Biogen Idec. **Dr**. **Razzolini** has received funding for travel and research support from Novartis. **Dr**. **Pozzilli** serves on scientific advisory boards for and has received speaker honoraria from Novartis, Merck Serono, Biogen Idec, Bayer Schering Pharma, and sanofi-aventis. **Dr**. **De Giglio** reports no disclosures. **Dr**. **Paolicelli** serves as a consultant for Merck Serono and Bayer Schering Pharma. **Dr**. **Trojano** has received speaker honoraria from Merck Serono, Bayer Schering Pharma, sanofi-aventis, and Biogen Idec; and has received research support from Biogen Idec and Merck Serono. **Dr**. **Marrosu** serves on scientific advisory boards for Merck Serono, Biogen Idec, and Bayer Schering Pharma; has received funding for travel from Biogen Idec, Merck Serono, Bayer Schering Pharma, and sanofi-aventis; serves on the editorial board of *Neurological Sciences*; has received speaker honoraria from Biogen Idec and Merck Serono; and has received research support from Merck Serono, Biogen Idec, and Fondazione Banco di Sardegna. **Dr**. **Patti** has served on scientific advisory boards for Merck Serono, Bayer Schering Pharma, Novartis, and Biogen Idec; has received speaker honoraria from Biogen Idec, Bayer Schering Pharma, sanofi-aventis, and Novartis; and has received research support from the University of Catania and FISM. **Dr**. **La Mantia** has received funding for travel from Biogen Idec and Bayer Schering Pharma. **Dr**. **Mancardi** has received funding for travel from Biogen Idec, Merck Serono, and Bayer Schering Pharma; serves on the editorial board of *Neurological Sciences*; and has received speaker honoraria from Biogen Idec and Bayer Schering Pharma. **Dr**. **Solaro** reports no disclosures. **Dr**. **Totaro** has received honoraria for consultancy or speaking from sanofi-aventis, Biogen Idec, Bayer Schering Pharma, and Merck Serono. **Dr**. **Tola** has served on scientific advisory boards for and received speaker honoraria from Biogen Idec, sanofi-aventis, Merck Serono, and Novartis; and has received research support from sanofi-aventis. **Dr**. **Di Tommaso** reports no disclosures. **Dr**. **Lugaresi** has served on scientific advisory boards for Biogen Idec, Merck Serono, and Bayer Schering Pharma; has received funding for travel and speaker honoraria from Bayer Schering Pharma, Biogen Idec, Merck Serono, Novartis, sanofi-aventis, and Teva Pharmaceutical Industries Ltd.; serves as a consultant for Fondazione "Cesare Serono"; and has received research support from Fondazione Italiana Sclerosi Multipla, Bayer Schering Pharma, Biogen Idec, Merck Serono, sanofi-aventis, Novartis, and AISM (Associazione Italiana Sclerosi Multipla). **Dr**. **Moiola** reports no disclosures. **Dr**. **Martinelli** has received funding for travel and speaker honoraria from Biogen Idec, Merck Serono, Bayer Schering Pharma, Novartis, and sanofi-aventis; and has served as a consultant to Bayer Schering Pharma, sanofi-aventis, and Teva Pharmaceutical Industries Ltd. **Dr**. **Comi** serves on scientific advisory boards for Bayer Schering Pharma, Merck Serono, Teva Pharmaceutical Industries Ltd., sanofi-aventis, Novartis, and Biogen Idec; and has received speaker honoraria from Teva Pharmaceutical Industries Ltd., sanofi-aventis, Serono Symposia International Foundation, Biogen Idec, Merck Serono, Novartis, and Bayer Schering Pharma.

**Dr**. **Amato** serves on scientific advisory boards for and has received speaker honoraria and research support from Biogen Idec, Merck Serono, Bayer Schering Pharma, and sanofi-aventis; and serves on the editorial board of *BMC Neurology*.

## Authors’ contributions

**LP**: Study concept or design. Acquisition of data. **EP**: Drafting/revising the manuscript. Study concept or design. Analysis or interpretation of data. Acquisition of data. Statistical analysis. Study supervision. **AG**: Drafting/revising the manuscript. Study concept or design. Acquisition of data. **BH**: Study concept or design. Acquisition of data. **MG**: Drafting/revising the manuscript. Study concept or design. Analysis or interpretation of data. Acquisition of data. **LR**: Study concept or design. Acquisition of data. **LDG**: Analysis or interpretation of data. Acquisition of data. **CP**: Drafting/revising the manuscript. Study supervision. **DP**: Analysis or interpretation of data. Acquisition of data. **MT**: Drafting/revising the manuscript. Study concept or design. Acquisition of data. **MGM**: Drafting/revising the manuscript. Acquisition of data. **FP**: Drafting/revising the manuscript. Study concept or design. Analysis or interpretation of data. Acquisition of data. **LLM**: Drafting/revising the manuscript. Acquisition of data. **GLM**: Study concept or design. Acquisition of data. **CS**: Analysis or interpretation of data. Study supervision. **RT**: Drafting/revising the manuscript. Acquisition of data. **MRT**: Drafting/revising the manuscript. Study concept or design. Acquisition of data. **VDT**: Analysis or interpretation of data. Acquisition of data. **AL**: Drafting/revising the manuscript. Analysis or interpretation of data. Acquisition of data. **LM**: Analysis or interpretation of data. Acquisition of data. **VM**: Drafting/revising the manuscript. Analysis or interpretation of data. Acquisition of data. **GC**: Drafting/revising the manuscript. Study supervision. **MPA**: Drafting/revising the manuscript. Study concept or design. Analysis or interpretation of data. Study supervision. All authors read and approved the final manuscript.

## Pre-publication history

The pre-publication history for this paper can be accessed here:

http://www.biomedcentral.com/1471-2377/12/165/prepub

## Supplementary Material

Additional file 1 Study interview. Click here for file

## References

[B1] Confavreux C Hutchinson M Hours MM Cortinovis-Tourniaire P Moreau T Rate of pregnancy-related relapse in multiple sclerosis. Pregnancy in Multiple Sclerosis Group N Engl J Med 1998 339 5 285 291 10.1056/NEJM199807303390501 9682040

[B2] Vukusic S Hutchinson M Hours M Moreau T Cortinovis-Tourniaire P Adeleine P Pregnancy and multiple sclerosis (the PRIMS study): clinical predictors of post-partum relapse Brain 2004 127 Pt 6 1353 1360 15130950 10.1093/brain/awh152

[B3] Langer-Gould A Huang SM Gupta R Leimpeter AD Greenwood E Albers KB Exclusive breastfeeding and the risk of postpartum relapses in women with multiple sclerosis Arch Neurol 2009 66 8 958 963 19506118 10.1001/archneurol.2009.132 PMC9622216

[B4] Langer-Gould A Gupta R Huang S Hagan A Atkuri K Leimpeter AD Interferon-gamma-producing T cells, pregnancy, and postpartum relapses of multiple sclerosis Arch Neurol 2010 67 1 51 57 10.1001/archneurol.2009.304 20065129 PMC9622213

[B5] Amato MP Portaccio E Ghezzi A Hakiki B Zipoli V Martinelli V Pregnancy and fetal outcomes after interferon-beta exposure in multiple sclerosis Neurology 2010 75 20 1794 1802 10.1212/WNL.0b013e3181fd62bb 21079181

[B6] Airas L Jalkanen A Alanen A Pirttila T Marttila RJ Breast-feeding, postpartum and prepregnancy disease activity in multiple sclerosis Neurology 2011 75 5 474 476 20679640 10.1212/WNL.0b013e3181eb5860

[B7] Portaccio E Ghezzi A Hakiki B Martinelli V Moiola L Patti F Breastfeeding is not related to postpartum relapses in multiple sclerosis Neurology Jul 12 77 2 145 150 21734184 10.1212/WNL.0b013e318224afc9

[B8] Vukusic S Confavreux C Pregnancy and multiple sclerosis: the children of PRIMS Clin Neurol Neurosurg 2006 108 3 266 270 10.1016/j.clineuro.2005.11.016 16413965

[B9] McDonald WI Compston A Edan G Goodkin D Hartung HP Lublin FD Recommended diagnostic criteria for multiple sclerosis: guidelines from the International Panel on the diagnosis of multiple sclerosis Ann Neurol 2001 50 1 121 127 10.1002/ana.1032 11456302

[B10] Kurtzke JF Rating neurologic impairment in multiple sclerosis: an expanded disability status scale (EDSS) Neurology 1983 33 11 1444 1452 10.1212/WNL.33.11.1444 6685237

[B11] Bader AM Hunt CO Datta S Naulty JS Ostheimer GW Anesthesia for the obstetric patient with multiple sclerosis J Clin Anesth 1988 1 1 21 24 10.1016/0952-8180(88)90006-2 3272740

[B12] Ferrero S Pretta S Ragni N Multiple sclerosis: management issues during pregnancy Eur J Obstet Gynecol Reprod Biol 2004 115 1 3 9 10.1016/j.ejogrb.2003.10.020 15223156

