# Supplementary material for: Epidural analgesia and cesarean delivery in multiple sclerosis post-partum relapses: the Italian cohort study
Source: BMC Neurol. 2012 Dec 31;12:165. doi: 10.1186/1471-2377-12-165 (PMC3544735; doi:10.1186/1471-2377-12-165)
Supplement: Additional file 1 — Study interview. [file 1471-2377-12-165-S1.doc]

**Appendix A: Study Interview**

Dati clinici

| Data di nascita (gg / m/aa) |  |
| --- | --- |
| Data di esordio SM (gg / m/aa) |  |
| Decorso SM (all’intervista) | RR  SP  PP |
| Menarca (età) |  |
| Scolarità (anni) |  |
| Fumo prima della gravidanza | No  Si_____________________________________ |
| Alcol prima della gravidanza | No  Si_____________________________________ |
| Familiarità per malformazioni | No  Si ____________________________________ |
| Familiarità per patologie significative | No  Si ____________________________________ |

**Disease modifying drugs** (in ordine di somministrazione)

| **Nome commerciale** | **Posologia** | **Inizio** | **Fine** | **Motivo della sospensione** |
| --- | --- | --- | --- | --- |
|  |  |  |  | Effetti collaterali  Inefficacia  Decisione del paziente  Programmata gravidanza  Gravidanza in atto |
|  |  |  |  | Effetti collaterali  Inefficacia  Decisione del paziente  Programmata gravidanza  Gravidanza in atto |
|  |  |  |  | Effetti collaterali  Inefficacia  Decisione del paziente  Programmata gravidanza  Gravidanza in atto |
|  |  |  |  | Effetti collaterali  Inefficacia  Decisione del paziente  Programmata gravidanza  Gravidanza in atto |
|  |  |  |  | Effetti collaterali  Inefficacia  Decisione del paziente  Programmata gravidanza  Gravidanza in atto |

**Informazioni sul ciclo mestruale**

| Alterazioni mestruali (prima o durante il trattamento) | No  Si  Prima del trattamento  Durante il trattamento con DMD (specificare quale farmaco ) |
| --- | --- |
| Metodo contraccettivo | No  Si  Prima del trattamento (specificare quale metodo contaccettivo)  Durante il trattamento con DMD (specificare quale metodo contaccettivo) |

# Gravidanza SM

| Inizio della gravidanza (data del 1° giorno dell’ultima mestruazione) |  |
| --- | --- |
| Assunzione di farmaci  I trimestre  II trimestre  III trimestre | No  Si (specificare)  No  Si (specificare)  No  Si (specificare) |
| Assunzione di acido folico | No  Si |
| Esposizione a radiazioni | No  Si (specificare trimestre) |
| Esposizione a tossici | No  Si (specificare quale e trimestre) |
| Esposizione a fumo | No  Si quantitativo/die trimestre |
| Esposizione ad alcol | No  Si quantitativo/die trimestre |
| Numero di feti |  |
| Aborto | No  Si  Epoca gestazionale ______________________  Causa accertata _________________________  Indagini eseguite sul feto _________________ |
| Complicazioni durante la gravidanza | NoSi(Specificare) __________________________________________ |
| Alterazioni ecografiche | NoSi(Specificare) __________________________________________ |
| Amniocentesi | Non eseguita  Eseguita (eventuali anomalie)  __________________________________________ |
| Data del parto (gg/m/aa) |  |
| Tipo di parto | vaginale  cesareo |
| Uso di strumenti durante il parto (forcipe, ventosa) | No  Si |
| Anestesia epidurale | No  Si |
| Complicazioni postpartum materne | No  Si (Specificare) _______________________ |

**Gravidanza SM**

**Outcomes fetali (compilare una scheda per ogni feto)**

| Settimane di gestazione |  |
| --- | --- |
| Sesso | M  F |
| Peso alla nascita (grammi) |  |
| Lunghezza alla nascita (cm.) |  |
| Apgar al 5° minuto |  |
| Complicazioni neonatali | No  Si (Specificare) __________________________________________ |
| Malformazioni | No  Si (Specificare) __________________________________________ |
| Morte | No  Si |

Allattamento

| Materno | Durata (mesi) |
| --- | --- |
| Misto | Durata (mesi) |
| Artificiale | Durata (mesi) |

**Età attuale del bambino ______________**

**Anomalie dello sviluppo psico-motorio**

No

Si (Specificare) __________________________________________

Attività di malattia

| Numero di ricadute nei 12 mesi precedenti la gravidanza |  |
| --- | --- |
| Data dell’ultima ricadute prima della gravidanza |  |
| Ricadute in gravidanza (indicare la data; specificare posologia, via di somministrazione e durata dell’eventuale trattamento steroideo) |  |
| Ricadute nei 12 mesi successivi al parto (data) |  |
| EDSS all’inizio della gravidanza |  |
| EDSS a 6 mesi dal parto |  |
| EDSS a 12 mesi dal parto |  |
| EDSS a 24 mesi dal parto |  |
